# Stress Distribution on Endodontically Treated Anterior Teeth Restored via Different Ceramic Materials with Varying Post Lengths Versus Endocrown—A 3D Finite Element Analysis

**DOI:** 10.3390/jfb16060221

**Published:** 2025-06-12

**Authors:** Mai Soliman, Nawaf Almutairi, Ali Alenezi, Raya Alenezi, Amal Abdallah A. Abo-Elmagd, Manal M. Abdelhafeez

**Affiliations:** 1 Department of Clinical Dental Sciences, College of Dentistry, Princess Nourah bint Abdulrahman University, Riyadh 11671, Saudi Arabia; 2 Department of Conservative Dental Sciences, College of Dentistry, Qassim University, Buraydah 52571, Saudi Arabia; nawaf.almutairi@qu.edu.sa (N.A.); m.abdelhafeez@qu.edu.sa (M.M.A.); 3 Department of Prosthetic Dental Sciences, College of Dentistry, Qassim University, Buraydah 52571, Saudi Arabia (A.A.); a.aboelmagd@qu.edu.sa or amal.abdallah4660@gmail.com (A.A.A.A.-E.); 4 General Dentistry, College of Dentistry, Qassim University, Buraydah 52571, Saudi Arabia; 5 Department of Fixed Prosthodontics, Faculty of Dental Surgery, MUST University, 6th of October City, Giza 12566, Egypt

**Keywords:** FEA, stress distribution, fiber post, post length, endocrown, Vita Enamic, Celtra Duo

## Abstract

Objective: This study aims to evaluate the stress distribution on endodontically treated anterior teeth restored using different restorative materials and different post lengths versus endocrowns employing finite element analysis (FEA). Methods: An extracted human central incisor tooth with a fully formed apex was scanned using high-resolution cone beam computed tomography (CBCT) to generate 3D finite element models. Six models of restorations of badly destructed central incisor were grouped according to the type of ceramic material and post length versus endocrown restorations. Group V-L: Vita Enamic, long post (10 mm intra-radicular), Group C-L: Celtra Duo, long post (10 mm intra-radicular), Group V-Sh: Vita Enamic, short post (3 mm intra-radicular), Group C-Sh: Celtra Duo, short post (3 mm intra-radicular), Group V-E: Vita Enamic endocrown (3 mm intra-radicular), and Group C-E: Celtra Duo endocrown (3 mm intra-radicular). A static load of 200 N was applied to the palatal surface at a 45 degree angle to the tooth’s long axis. The maximum equivalent von Mises stress and maximum principal stress were analyzed at four locations: the finish line, coronal third of the root (12 mm from the apex), middle third of the root (8 mm from the apex), and apical third of the root (4 mm from the apex). Results: Group C-L exhibited the highest maximum VM stress and PS at the finish line, in addition to the highest maximum VM stress and PS at the root apical third, while group C-Sh reported the least maximum VM stress at the root apical third among the groups. All Celtra Duo groups reported higher maximum VM stress than the corresponding groups of Vita Enamic at the finish line and root coronal thirds. However, at the root middle and apical thirds, both materials recorded similar stresses. Conclusions: Short posts and Vita Enamic endocrowns showed minimal stress, especially at the finish line, while long posts increased stress and fracture risk. The findings support conservative restorations without posts, although clinical validation is needed to confirm their long-term effectiveness and safety.

## 1. Introduction

Endodontically treated teeth are inherently more susceptible to structural damage and fractures compared to vital teeth. This increased risk of fracture is primarily due to loss of tooth structure during endodontic therapy and subsequent restorative procedures, which can compromise the tooth’s integrity and biomechanical properties. The presence of a post and core, along with various restorative prostheses, plays a crucial role in the prognosis and durability of these treated teeth [1,2,3,4,5].

Finite element analysis (FEA) is a computational tool that simulates the behavior of structures under various conditions. It employs the finite element method (FEM) to divide a structure into small, discrete elements and solve the equations of equilibrium for each element. This method allows for the detailed analysis of stress distribution, identifying potential weak spots and design flaws [6]. FEA has been successfully adopted in various engineering fields and has proven to be particularly beneficial in the field of dentistry for studying the biomechanical behaviors of dental structures and materials [7,8].

The application of FEA in dentistry has provided valuable insights into the stress distribution and fracture resistance of endodontically treated teeth restored with different materials and techniques. Studies have shown that the type of post, core, and crown material, as well as the length and design of the post, significantly affect the stress distribution within the restored tooth [9]. Proper selection of these components is essential to enhance the longevity and performance of the restoration.

Endodontically treated teeth often require the placement of a post to retain the core material and provide adequate support for the final restoration. The post and core system compensates for the loss of tooth structure and helps to distribute occlusal forces along the length of the root, reducing the risk of fracture [10,11,12]. Various materials are used for posts, including metal, fiber-reinforced composite, and ceramic, each with its own biomechanical properties and clinical implications [13,14,15,16].

FEA has become an indispensable tool in evaluating the biomechanical performance of various post, core, and crown materials used in the restoration of endodontically treated teeth. The selection of these materials significantly influences stress distribution within the tooth structure, thereby affecting the longevity and success of dental restorations [17].

Core materials are essential in reinforcing structurally compromised teeth, particularly following endodontic treatment. Their mechanical properties, notably the elastic modulus, significantly influence stress distribution within the tooth–restoration complex. FEA studies have demonstrated that composite resin cores, due to their elastic modulus being closer to that of dentin, facilitate a more uniform stress distribution. This compatibility reduces the risk of stress concentrations that could lead to structural failures. For instance, a study by Ouldyerou et al. (2023) [17] revealed that increasing the Young’s modulus of composite materials from 10 GPa to 26 GPa resulted in decreased stress levels in both enamel and dentin under occlusal loading, highlighting the importance of selecting core materials with mechanical properties akin to natural tooth structures [17].

By contrast, metallic cores, such as cast gold alloys, possess a significantly higher elastic modulus compared to dentin, leading to increased stress concentrations at the core–dentin interface. This disparity can result in unfavorable stress distributions, potentially compromising the integrity of the tooth [17].

Fiber posts have gained popularity due to their favorable biomechanical properties, such as a modulus of elasticity similar to dentin, which helps in reducing stress concentrations and improving fracture resistance [9,18,19]. However, the effectiveness of fiber posts depends on several factors, including the length of the post, the type of core material, and the design of the final restoration. The choice of crown material also plays a crucial role in the overall performance of the restored tooth [20,21,22,23].

The elastic modulus of post materials plays a crucial role in stress distribution. Materials with an elastic modulus closer to that of dentin, such as glass fiber posts, have been shown to produce more homogeneous stress distributions, potentially reducing the risk of root fractures [24]. Conversely, stiffer materials like cobalt-chromium can lead to higher stress concentrations, particularly in areas with complex root canal morphologies. This underscores the importance of material selection based on the specific anatomical considerations of the tooth being restored [25].

Vita Enamic and Celtra Duo are two widely used materials for dental crowns. Vita Enamic is a hybrid ceramic material that combines the properties of ceramic and composite, offering high strength, durability, and esthetics. Celtra Duo is a zirconia-reinforced lithium silicate ceramic known for its excellent mechanical properties and esthetic qualities [26]. Both materials have been extensively studied for their performance in dental restorations, but their impact on stress distribution in endodontically treated teeth requires further investigation [27].

Crown materials also significantly impact stress distribution. A study by Lin, J. et al. (2020) [23] compared zirconia and lithium disilicate-reinforced glass ceramic crowns, finding that zirconia crowns exhibited higher stress concentrations in the crown structure, while lithium disilicate crowns showed more favorable stress distributions in the underlying tooth structure. These findings highlight the need for a comprehensive approach in selecting crown materials, considering both the mechanical properties of the materials and their interaction with the tooth–restoration complex [23].

In addition to material properties, the design of the restoration, particularly the length of the post, has a significant impact on the stress distribution within the tooth. Longer posts are generally believed to provide better retention and stability for the core and crown, but they can also introduce higher stress concentrations at the apical end, potentially leading to root fractures [28,29,30,31]. Conversely, shorter posts may reduce the risk of apical stress but may not provide sufficient support for the restoration, especially in teeth with extensive structural loss [32].

The concept of endocrowns, which integrate the core and crown into a single restoration with an intra-radicular extension, has gained attention as an alternative approach for restoring endodontically treated teeth. Endocrowns are designed to distribute occlusal forces more evenly and minimize stress concentrations at critical points within the tooth structure [33,34,35]. Studies have shown that endocrowns can be particularly effective in restoring molars and premolars, but their application in anterior teeth requires further exploration [36,37,38].

This study aims to evaluate the stress distribution on endodontically treated anterior teeth using different restoration materials with variable post lengths through FEA. By comparing the performance of Vita Enamic and Celtra Duo crowns, with both long and short posts, as well as endocrowns with 3 mm intra-radicular extensions, this study seeks to identify the optimal restorative approach for enhancing the durability and prognosis of endodontically treated anterior teeth.

## 2. Materials and Methods

### 2.1. Model Construction

This in vitro study was granted ethical approval from the Committee of Research Ethics, Deanship of Scientific Research, Qassim University, under study number IRB 24-76-09. All methods were performed in accordance with the relevant guidelines and regulations. A fully developed human central incisor tooth with fully formed apex was scanned using high-resolution cone beam computed tomography (CBCT) (Planmeca ProMax 3D MID; Planmeca, Helsinki, Finland) operating at 90 kV and 12 mA with a voxel dimension of 75 mm, to obtain about 800 images. The images were processed using the Materialize Interactive Medical Image Control System (MIMICS 19.0; Materialise, Leuven, Belgium) to identify enamel and dentin tissues using voxel intensity and the mathematical region growing feature. A three-dimensional stereolithography (3D-STL) file format was then exported for both enamel and dentin structures, and the data were optimized using 3-Matic Medical 11.0 software (Materialise NV).

The file was imported into SolidWorks software 2018 (Dassault Systems, Vélizy-Villacoublay, France), where enamel and dentin were combined. The surrounding periodontal ligaments (PDL) were then established, extending from the cementoenamel junction (CEJ) to the apical portion of the root with a uniform thickness of 200 µm, and supported by alveolar bone 3 mm below the CEJ.

### 2.2. Restoration Modeling and Grouping (Table 1 and Table 2)

Six three-dimensional finite element models were created, with each representing a restorative scenario:

- Group V-L: Vita Enamic crown—Long post; 10 mm intra-radicular length, and composite core.
- Group C-L: Celtra Duo crown—Long post; 10 mm intra-radicular length, and composite core.
- Group V-Sh: Vita Enamic crown—Short post; 3 mm intra-radicular length, and composite core.
- Group C-Sh: Celtra Duo crown—Short post; 3 mm intra-radicular length, and composite core.
- Group V-E: Vita Enamic—Endocrown with 3 mm intra-radicular extension.
- Group C-E: Celtra Duo—Endocrown with 3 mm intra-radicular extension.

### 2.3. Model Preparation

The coronal portion of the created models was prepared to simulate a badly broken central incisor, leaving 2.0 mm ferrule coronal to the cementoenamel junction (CEJ) and a 0.5 mm chamfer finish line.

The root canal was prepared following the dimensions of the Protaper Next (PTN) rotary filing system (Dentsply; Maillefer, Ballaigu, Switzerland) with an estimated size X3: 30/0.07 taper and shaped to a centralized conical dimension. Afterward, the canal was obturated using gutta-percha points size X3 of the same PTN rotary filling system, together with Bioceramic sealer (Brasseler USA, Savannah, GA, USA), starting 0.5 mm from the apex and extending coronally.

The gutta-percha was then seared to the desired lengths according to the estimated groups. As for the groups using long posts [Group V-L and C-L], 10 mm of the gutta-percha was removed coronally. However, for the groups using short posts [Group V-Sh and C-Sh], only 3 mm of gutta-percha was removed coronally from the canal. For the endocrown restorations [Group V-E and C-E], 3 mm of the gutta-percha was removed coronally as well.

**Figure 1:**
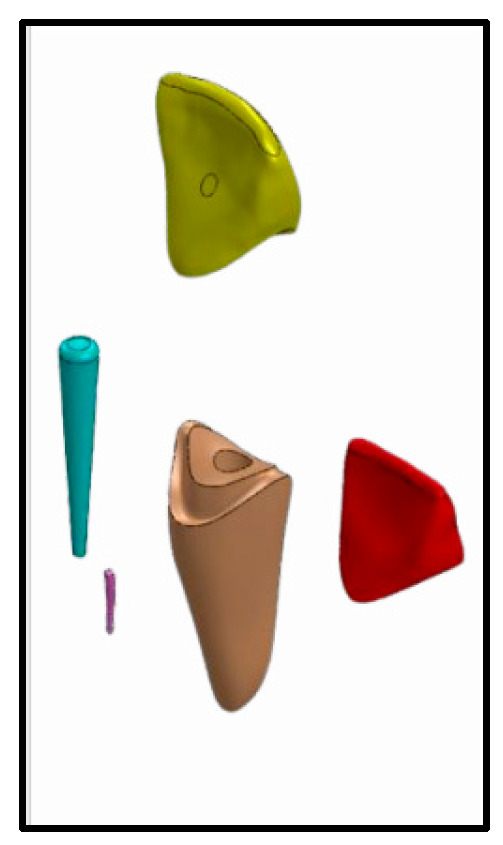
Model for Group V-L and Group C-L; Vita Enamic/Celtra Duo crown, Long post; 10 mm intra-radicular length, and composite core. 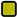 crown, 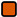 composite core, 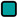 fiber post, 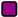 gutta-percha, 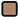 tooth structure.

For the groups using long posts [Group V-L and C-L], a size 1/yellow drill was used for post space preparation, then size 1/yellow fiber post (3M™ RelyX™ Fiber Post 3D Glass Fiber Post, 3M Canada, London, ON, Canada) was cemented using self-adhesive resin cement (3M™ RelyX™ Unicem 2 Automix, 3M Canada, Canada) (Figure 1).

**Figure 2:**
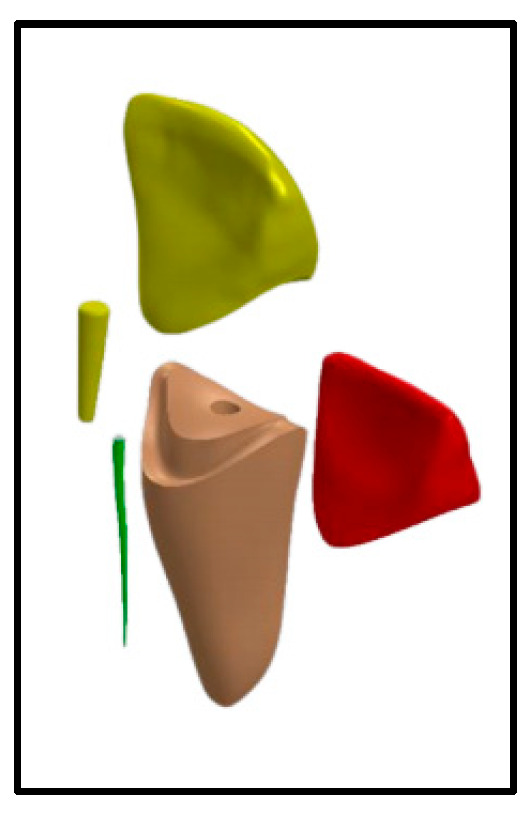
Model for Group V-Sh and Group C-Sh; Vita Enamic/Celtra Duo crown, Short post; 3 mm intra-radicular length, and composite core. 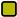 crown, 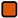 composite core, 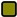 fiber post, 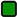 gutta-percha, 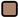 tooth structure.

For the groups using short posts [Group V-Sh and C-Sh], a size 2/red drill was used for post space preparation, then size 2/red fiber post (3M™ RelyX™ Fiber Post 3D Glass Fiber Post, 3M Canada, London, ON, Canada) was cemented using self-adhesive resin cement (3M™ RelyX™ Unicem 2 Automix, 3M Canada, London, ON, Canada) (Figure 2).

For both the short and long post groups [Group V-L, C-L, V-Sh, and C-Sh], adhesive for core build up (3M™ Scotchbond™ Universal Adhesive, 3M Canada, Canada) and core build-up material (3M™ Filtek™ One Bulk Fill Restorative, 3M Canada, Canada) were used to build the composite core according to manufacturer’s instructions. Composite cores were prepared to receive ceramic crowns that were fabricated using Vita Enamic (Vita, Vita Zahnfabrik, Bad Säckingen, Germany) and Celtra Duo (Dentsply, Sirona, Dental Systems Gmbh FabrikstraBe, Bensheim, Germany), respectively (Figure 1 and Figure 2).

**Figure 3:**
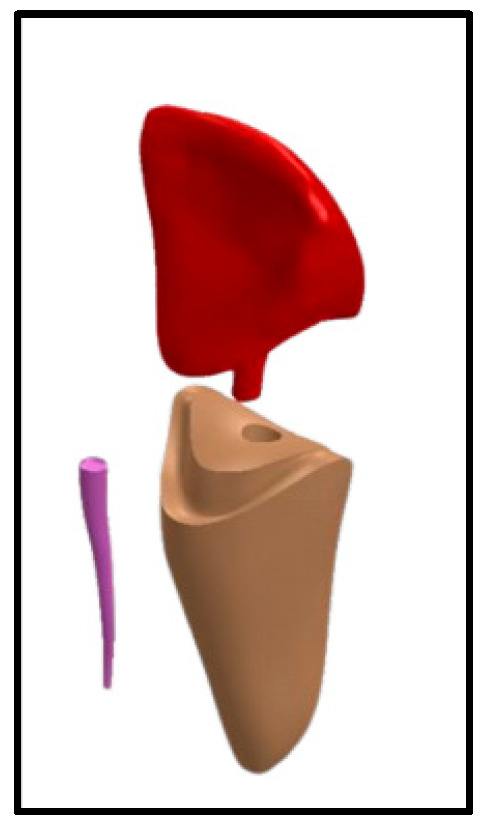
Model for Group V-E and Group C-E: Vita Enamic/Celtra Duo Endocrown with 3 mm intra-radicular extension. 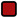 Endocrown, 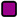 gutta-percha, 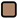 tooth structure.

For the groups restored by endocrown [Group V-E and C-E], a size 2/red post drill was used for post space preparation, the endocrowns were CAD/CAM-fabricated using Vita Enamic (Vita, Vita Zahnfabrik, Bad Säckingen, Germany) and Celtra Duo (Dentsply, Sirona, Dental Systems Gmbh FabrikstraBe, Bensheim, Germany), respectively, and then finished and polished according to the manufacturer’s instruction (Figure 3).

The ceramic crowns and endocrowns were finally cemented on their respective models using self-adhesive resin cement (3M™ RelyX™ Unicem 2 Automix, 3M Canada, Canada).

#### Boundary Conditions and Load Application

**Figure 4:**
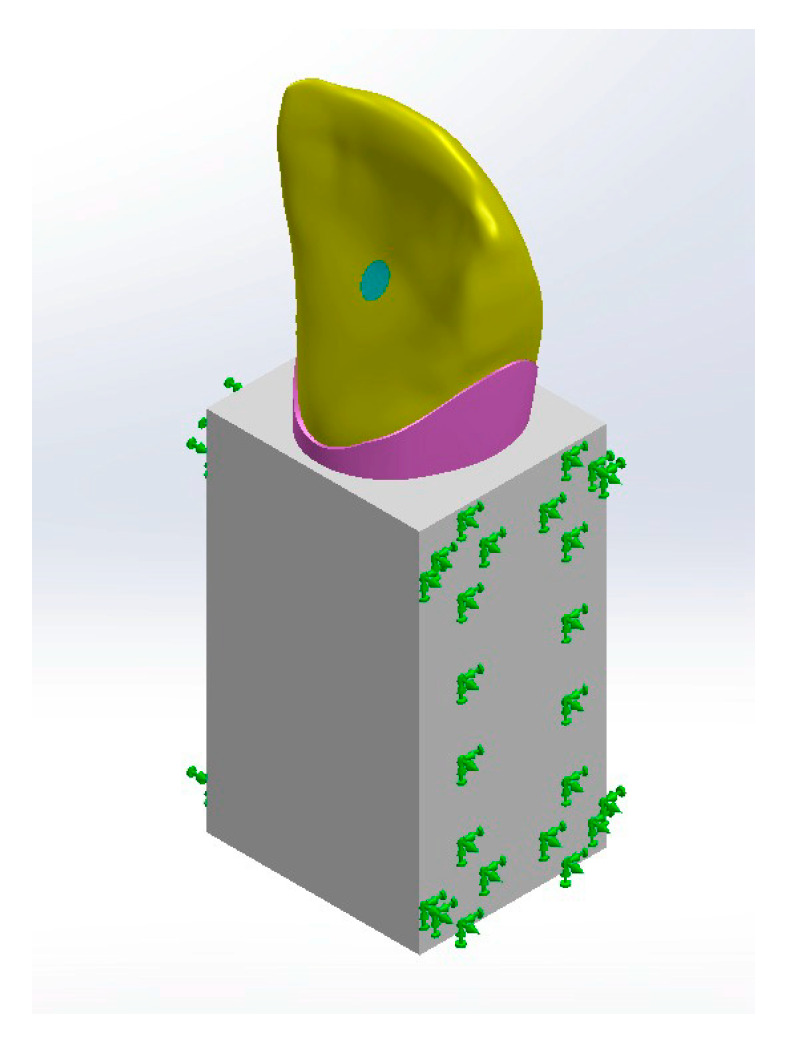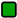 Boundary conditions and 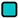 load application.

All bodies were simulated to be bonded to restrict the movement of a specific node or group of nodes within the finite element model. This constraint simulated a real-world situation where a physical object is fixed or attached to a stationary surface or structure, preventing it from moving freely in one or more directions. Boundary bonding means “no body is allowed to move relative to the body touching it” to any touching body. The cancellous block was fixed at both mesial and distal surfaces to isolate the region of interest (ROI) from outer effects (Figure 4).

The 3D models of the maxillary central incisor were subjected to a static load of 200 N magnitude directed on the lingual surface at a 45 degree angle to the tooth axis [39,40]. The evaluation of von Mises (VM) stress and principal stress (PS) focused on the pattern of stress distribution, and the maximum values were observed (Figure 4).

The magnitudes of the VM stress and PS were observed at four locations: the finish line, coronal third of the root (12 mm from the apex), middle third of the root (8 mm from the apex), and apical third of the root (4 mm from the apex) of the models [41,42]. Stress distribution was analyzed as colorimetric maps, with adjustable color scales corresponding to stress magnitude comparisons among the designs for each analyzed group.

**Figure 5:**
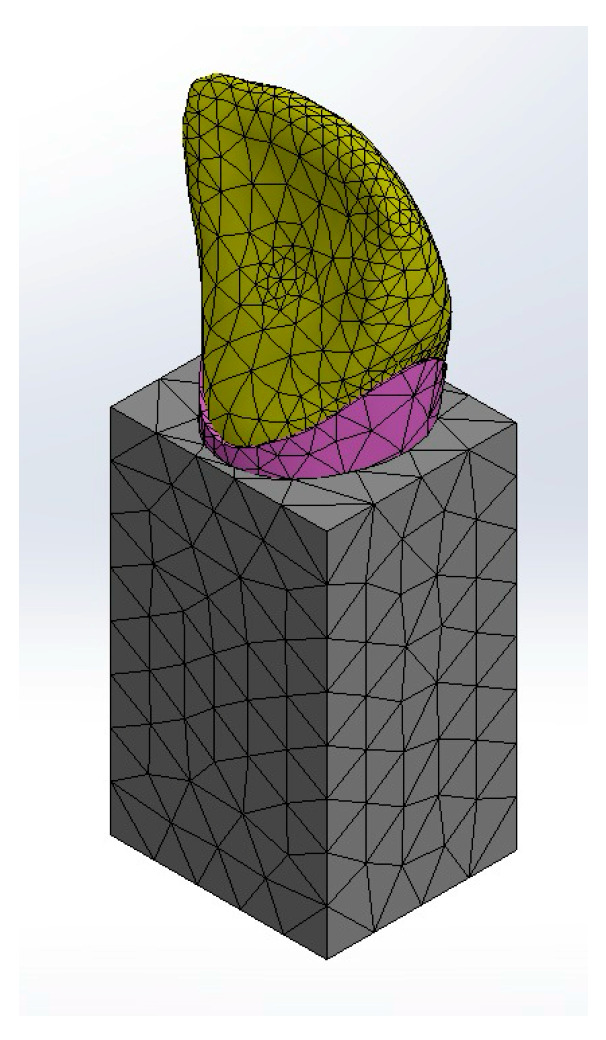
Mesh of the models.

The 3 models were meshed with six degrees of freedom per node: (Figure 5).

- For long post groups (V-L & C-L): 13,108 quadratic tetrahedral elements and 23,169 nodes.
- For short post groups (V-Sh& C-Sh): 15,705 quadratic tetrahedral elements and 26,733 nodes.
- For Endocrown groups (V-E & C-E): 14,037 quadratic tetrahedral elements and 24,084 nodes

**Figure 6:**
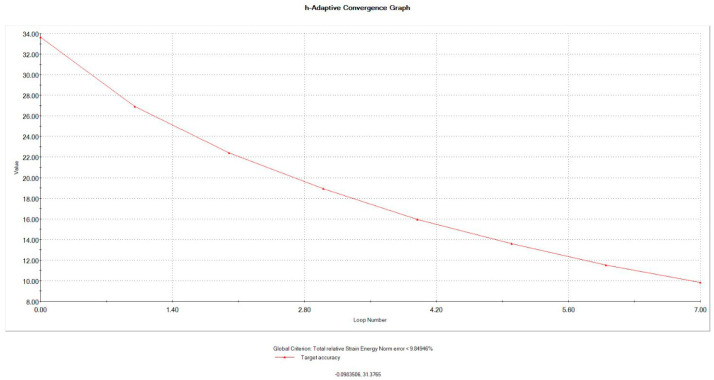
Mesh h-adaptive convergence graph.

The behaviors of the meshed models during stress application as the real situation were validated by mesh convergence analysis to calculate the error percentage to reduce the error to the minimum acceptable error, which was considered to be 3% in our study (Figure 6).

The material properties used in the FEA models, including various elastic moduli and Poisson’s ratios for dentin, cementum, cancellous bone, periodontal ligament, gutta-percha, composite resin, adhesive resin cement, Vita Enamic, Celtra Duo, and fiber posts, are detailed in Table 3.

## 3. Results

**Figure 7:**
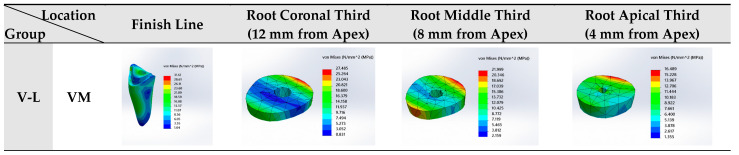

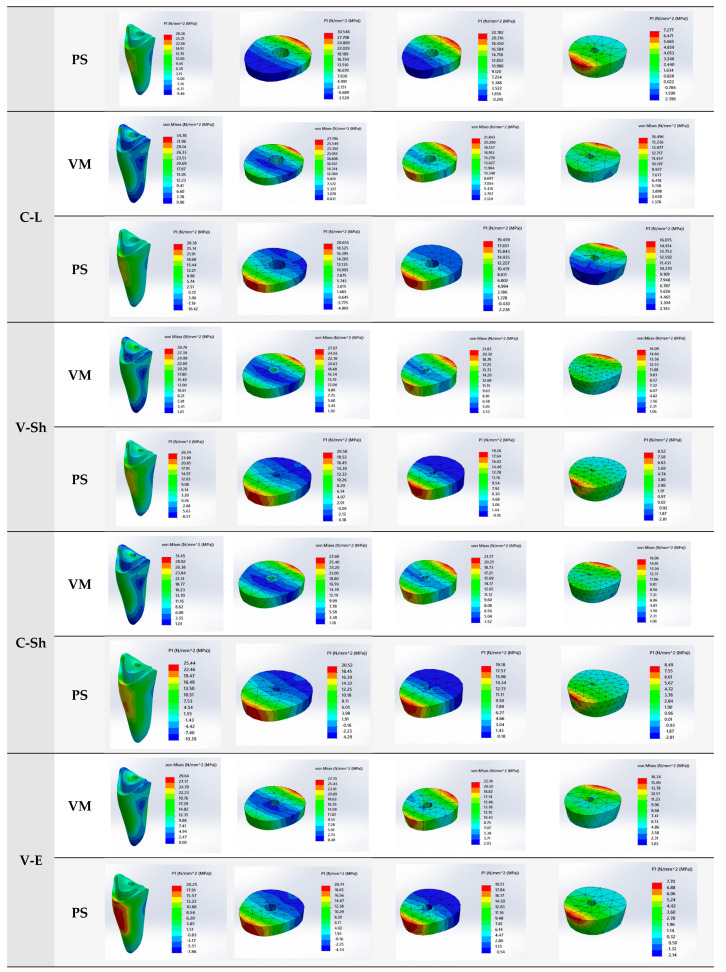

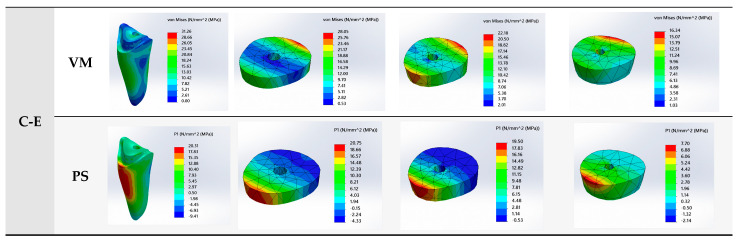
Von Mises (VM) stress distribution and principal stress (PS) at finish line and different root sections for each group.

Among all tested groups, Group C-L exhibited the highest values of maximum VM stress and PS at the finish line location (34.78 MPa and 28.38 MPa, respectively), in addition to the highest values of maximum VM stress and PS at the root apical third (16.496 MPa and 16.075 MPa, respectively), while group C-Sh reported the least VM stress at the root apical third among the groups (16.06 MPa) (Table 4, Figure 7).

Regarding finish line location, Group C-L exhibited the highest values of maximum VM stress and PS at the finish line (34.78 MPa and 28.38 MPa, respectively), while group V-E reported the least values of maximum VM stress and PS among the groups (29.64 MPa and 20.25 MPa, respectively) (Table 4, Figure 7).

Regarding coronal third location of the root (12 mm from the apex), Group C-E showed the highest value of maximum VM stress (28.05 MPa), group V-L showed the highest value of maximum PS (30.548 MPa), while group V-Sh displayed the least value of VM stress among the groups (27.07 MPa) (Table 4, Figure 7).

Regarding middle third location of the root (8 mm from the apex), both the V-E and C-E groups showed the highest value of maximum VM stress at the root middle third (22.18 MPa), group V-L showed the highest value of maximum PS (22.182 MPa), while group C-Sh displayed the least values of maximum VM stress and PS among the groups (21.77 MPa and 19.18 MPa, respectively) (Table 4, Figure 7).

Regarding apical third location of the root (4 mm from the apex), Group C-L exhibited the highest values of maximum VM stress and PS at the root apical third (16.496 MPa and 16.075 MPa, respectively), while group C-Sh reported the least value of maximum VM stress among the groups (16.06 MPa) (Table 4, Figure 7).

The three Celtra Duo groups reported higher VM stress than the corresponding groups of Vita Enamic at the finish line and root coronal thirds. However, at the root middle and apical thirds, both materials recorded similar value of VM stress (Table 4, Figure 7).

## 4. Discussion

The current study employed three-dimensional FEA to evaluate the stress distribution on endodontically treated anterior teeth restored using different restorative options, crown materials, and post lengths. This method allowed the quantification and characterization of the stress distribution after having applied external functional forces [50]. The findings from this study provide significant insights into the biomechanical performance of various restorative options, which are crucial for optimizing the prognosis and longevity of endodontically treated teeth.

While laboratory tests yield valuable insights into tooth fracture, they typically rely on destructive methods and cannot fully capture the stress–strain behavior of dental restorations [7,8,9,10,11,12,13,14,15,16,17,18,19,20,21,22,23,24,25,26,27,28,29,30,31,32,33,34,35,36,37,38,39,40,41,42,43,44,45,46,47,48,49,50,51]. Finite element analysis (FEA), on the other hand, is a powerful, non-destructive tool widely used in dental research to evaluate stress distribution and mechanical behavior within dental structures. By constructing a geometric model and subdividing it into smaller elements, FEA applies mathematical equations to simulate how the structure responds to various forces. This method is highly accurate and capable of modeling complex anatomical structures, such as teeth and their supporting tissues, in detail. Three-dimensional FEA (3D FEA) is especially prevalent, offering detailed simulations based on the mechanical properties of materials [52,53,54,55]. However, it is important to note that FEA cannot account for certain factors influencing restoration performance, including microleakage, polymerization shrinkage, and postoperative sensitivity [56].

Although dental tissues possess anisotropic properties, their elastic modulus shows only minimal variations in different directions under applied forces [57]. Similarly, the periodontal ligament exhibits negligible differences in stress distribution when modeled with either linear or nonlinear force responses. Therefore, like many other studies, the current research assumed that all materials are isotropic, linearly elastic, and homogeneous as a practical simplification [58,59]. All interfaces were also modeled as fully bonded, without any gaps or voids.

Endodontically treated teeth exhibit a high risk of biomechanical failure due to loss of tooth structure by dental trauma or caries. Therefore, their rehabilitation is considered as a clinical challenge [1]. They can be restored by post–core, crown, inlay only, and endocrown. Endocrown can be defined as an adhesive restoration that has a single unit of core and crown. The advantages of endocrown restorations are that it is a conservative technique, is less time consuming, and reduces treatment costs. It is indicated to restore endodontically treated teeth with anatomically variated roots, calcified, or curved root canals that would hinder the placement of regular post–core and with teeth that have extensive loss of coronal structure [60].

This study examines two CAD/CAM ceramic materials: Celtra Duo, a zirconia-reinforced lithium silicate (ZLS) containing 10% zirconium dioxide, which enhances strength while maintaining machinability for crowns, inlays, onlays, and veneers; and Vita Enamic, a polymer-infiltrated ceramic network (PICN) with 75% glass ceramic and 25% polymer, which combines ceramic strength with polymer resilience, enabling precise milling, efficient finishing, and suitability for minimally invasive restorations [60].

In the current study, the glass fiber post system was used as it offers several advantages over metallic posts, which often generate high stresses and lead to non-restorable root fractures. With an elastic modulus similar to dentin, glass fiber posts improve bending resistance and absorb forces more effectively, reducing fracture risk. Unlike high-modulus posts, which increase the likelihood of severe fractures, glass fiber posts distribute stress more evenly, minimizing damage and allowing for easier restoration if failure occurs [61].

The role of post length is particularly important in determining the tooth’s flexural behavior and fracture resistance. In posterior teeth, where masticatory forces are primarily compressive, the post aids in core retention [62]. However, in anterior teeth, which experience transverse loading, post length can influence fracture resistance. Recent studies suggest that shortening the post length does not negatively affect fracture resistance or compromise the apical seal. With modern adhesive techniques reducing the need for macro retentive elements, radicular posts may no longer be essential in posterior teeth, although limited data exist on their necessity in anterior teeth [61].

Endocrowns offer a convenient and cost-effective restorative option for endodontically treated teeth. Compared to conventional crowns with short or long posts, they require less clinical time and simplify the process. By eliminating the post and filling core, endocrowns also reduce bonding interfaces to potentially enhance structural integrity. Moreover, endocrowns are designed to distribute occlusal forces more evenly and minimize stress concentrations at critical points within the tooth structure [61].

The results of the current study revealed that Group C-L exhibited the highest maximum VM stress and PS values at the finish line (34.78 MPa and 28.38 MPa, respectively), as well as the highest maximum VM stress and PS values at the root apical third (16.496 MPa and 16.075 MPa, respectively), while group C-Sh reported the least stress at the root apical third among the groups (16.06 MPa). The results indicated that long post groups transferred more stress to the apical region, while short post groups had the least stress transfer, with the endocrown groups showing intermediate values. Similar findings [28] highlighted that although longer posts enhance retention, they also concentrate stress at the apical third, potentially raising the risk of root fractures. These findings emphasize the need to balance post length with tooth integrity and crown material selection.

Similar results were reported by Cruzado-Oliva FH [63], who observed that the stress distribution in endocrowns without ferrules was significantly lower than in traditional restorations with crowns and posts, aligning with other studies [64,65,66]. This is because fewer material interfaces allow better stress distribution, as seen in the monolithic structure of endocrowns [67,68], which also reduces the cement layer. By contrast, crown posts require three to four layers of composite resin for customization; otherwise, a thick cement layer forms, increasing fracture risk [69] Additionally, if an endocrown-treated tooth fractures, the break typically occurs at the cervical region [52], allowing for potential repair through crown lengthening surgery or orthodontic traction, unlike post-core crown restorations [67,68,69,70,71].

In addition, the stress distribution in long posts may be influenced by depth, as greater depth leads to higher stress, consistent with findings from other studies [67,68,72], possibly due to an increased contact surface area [73].

The ferrule effect is well documented in enhancing the fracture resistance of endodontically treated teeth. Studies have shown that increasing the ferrule height can lead to improved stress distribution and mechanical stability. For example, a study by Sherfudhin et al. [74] demonstrated that different ferrule designs significantly affect the fracture resistance and failure patterns of restored teeth. Similarly, finite element analysis by Eraslan et al. [75] indicated that a 2 mm ferrule height reduced the stress concentration within the root structure. These findings suggest that variations in ferrule height can have a substantial impact on the biomechanical performance of restorations.

The results demonstrated that endocrowns provided favorable stress distribution patterns. For instance, at the finish line location, Group V-E (Vita Enamic endocrown) demonstrated a maximum stress of 29.64 MPa, which was lower than the stress observed in the groups with traditional post and core systems. Similarly, Group C-E (Celtra Duo endocrown) showed a maximum stress of 31.26 MPa at the finish line. These findings suggest that endocrowns can effectively distribute stress and potentially enhance the biomechanical performance of restorations in endodontically treated teeth [32].

Bindl and Mormann [33] reported similar findings, indicating that endocrowns are particularly effective in restoring molars due to their ability to distribute occlusal forces more evenly. Biacchi and Basting [32] further supported the use of endocrowns, demonstrating their superior fracture resistance compared to traditional post and core systems. Our study extends these findings to anterior teeth, highlighting the potential of endocrowns to enhance the durability and prognosis of restorations.

However, one study found that teeth restored with endocrowns and fiberglass-reinforced posts exhibited similar fracture strength, as endocrowns function like short posts [37]. By contrast, other studies suggest that the depth of endocrown preparation does not significantly affect fracture resistance [68] or stress distribution [66]. Therefore, restoring extensively damaged anterior teeth with an endocrown or a short glass fiber post may offer advantages over a longer glass fiber post [61].

Going through the effect of crown material on stress distribution, all Celtra Duo groups reported higher stresses than the corresponding groups of Vita Enamic at the finish line and root coronal thirds. However, at the root middle and apical thirds, both materials recorded similar stresses, and it was clearly observed that the choice of crown material significantly impacts the stress distribution in endodontically treated teeth.

These findings are in line with those reported by Heintze et al. [26], who found that hybrid ceramic materials like Vita Enamic provide better stress distribution and lower fracture risk compared to zirconia-reinforced ceramics. Another study conducted by Conserva et al. [76] reported that composite materials with a Young’s modulus similar to that of dentin should be selected for the restoration of endodontically-treated teeth when using carbon fiber posts. However, Kelly and Benetti [27] highlighted that the choice of crown material should also consider aesthetic requirements and patient-specific factors, suggesting that Celtra Duo’s superior aesthetics could make it a preferable choice in certain clinical scenarios despite its slightly higher stress concentrations.

The findings from this study have several important clinical implications. First, the choice of post length should be carefully considered based on the specific clinical scenario. While longer posts generally provide better retention, they can also introduce higher stress concentrations at the apical third, which may increase the risk of root fractures. Therefore, the decision on post length should be balanced with other factors, such as the type of crown material and the overall condition of the tooth.

Second, the choice of crown material is critical for optimizing the biomechanical performance of restorations. Vita Enamic crowns demonstrated lower stress concentrations at the finish line compared to Celtra Duo crowns, suggesting that they may be a better option for reducing the risk of fractures and enhancing the durability of the restoration. However, Celtra Duo crowns also performed well, particularly in endocrown configurations, indicating that they can be a viable option depending on the specific clinical requirements [1,10,28,77].

The use of endocrowns with a 3 mm intra-radicular extension offers a promising alternative to traditional post and core systems. The favorable stress distribution patterns observed in this study suggest that endocrowns can effectively minimize stress concentrations and enhance the biomechanical performance of restorations. This can be particularly beneficial in cases where there is significant structural loss or when preserving as much tooth structure as possible is a priority [9,10,36,38].

In spite of the fact that the finite element method is widely used due to its rapid mechanical analysis and versatility, it cannot yet fully simulate the complex forces generated in the oral cavity. It only evaluates ideal stress conditions under static loads, whereas real chewing involves dynamic forces. Factors such as the presence of periodontal disease, variations in bone density, and patient-specific occlusal forces were not considered. With advancements in finite element analysis technology, future studies may achieve more complex biomechanical simulations or include supporting in vitro studies of mechanical testing, which in turn will validate the finite analysis outcomes.

The current study focused on a specific tooth type to maintain controlled conditions and reduce variability. However, we recognize that anatomical differences between anterior and posterior teeth, such as root morphology, canal configuration, and loading patterns, can significantly influence stress distribution and fracture resistance. For instance, posterior teeth typically endure higher occlusal forces and have different structural characteristics compared to anterior teeth. Therefore, while our findings provide valuable insights, caution should be exercised when extrapolating these results to other tooth types. Future studies should aim to include a variety of tooth types to enhance the applicability of the results across different clinical scenarios.

Future studies should aim to validate these findings through clinical trials and in vivo studies to confirm the biomechanical performance of different restorative options under real-world conditions. Additionally, further research should explore the impact of other factors, such as tooth morphology, occlusal loading patterns, and the presence of adjacent teeth, on stress distribution. This will provide a more comprehensive understanding of the factors that influence the success and longevity of restorations in endodontically treated teeth.

## 5. Conclusions

Post length has a non-significant impact on stress distribution in endodontically treated anterior teeth. However, short posts generate the least stress at all root levels, while long posts increase stress at the finish line and apical third, raising the risk of fractures.

Endocrowns, especially those made from Vita Enamic, exhibit favorable stress distribution at the finish line, making them a strong alternative to traditional post and core systems. Their mechanical performance suggests they could replace long post restorations while preserving more tooth structure.

Restorations without posts offer the advantage of preserving more tooth substance and simplifying clinical procedures. This supports the use of endocrowns or short posts for anterior teeth as a viable restorative approach.

Although the study demonstrates the mechanical superiority of endocrowns and short posts, in vivo validation is necessary before this technique can be widely recommended for clinical use.

## Figures and Tables

**Table 1:** Model Grouping.

	Design	Long Post (10 mm Intra-Radicular)	Short Post (3 mm Intra-Radicular)	Endocrown (3 mm Intra-Radicular)
Material				
Vita Enamic		Group V-L	Group V-Sh	Group V-E
Celtra Duo		Group C-L	Group C-Sh	Group C-E

**Table 2:** The two tested materials’ compositions (Vita Enamic and Celta Duo).

Material	Manufacturer	Ceramic Type	Chemical Composition
Vita Enamic	VITA-Zahnfabrik, Bad Säckingen, Germany	Polymer-infiltrated ceramic	Polymer-infiltrated feldspathic ceramic network material (UDMA, TEGDMA) with 86 wt% ceramic (SiO_2_, Al_2_O_3_, Na_2_O, K_2_O, B_2_O_3_, CaO, TiO_2_, coloring oxides)
Celtra Duo	Dentsply, Charlotte, NC, USA	Zirconia-reinforced lithium silicate ceramic	SiO_2_, P_2_O_5_, Al_2_O_3_, Li_2_O, ZnO, 10% ZrO_2_

**Table 3:** Elastic moduli and Poisson’s ratios of the materials used in the model.

Material	Elastic Modulus (GPa)	Poisson’s Ratio
Dentin [43,44,45]	18.6	0.31
Cementum [44]	8.2	0.3
Cancellous bone [43,44,45,46,47]	1.37	0.3
Periodontal ligament [43,47]	0.05	0.45
Gutta percha [43]	0.14	0.45
Fiber post [43]	33	0.33
Composite resin [47,48]	16.6	0.24
Adhesive resin cement [43]	18.6	0.28
Vita Enamic [45]	30	0.23
Celtra Duo [49]	70	0.23

**Table 4:** Maximum Von Mises (VM) stress and principal stress (PS) values at different sections for each group.

	Location	Finish Line	Root Coronal Third (12 mm from Apex)	Root Middle Third (8 mm from Apex)	Root Apical Third (4 mm from Apex)
Group					
V-L	VM	31.12	27.485	21.999	16.489
	PS	28.36	30.548	22.182	7.277
C-L	VM	34.78	27.796	21.843	16.496
	PS	28.38	20.655	19.459	16.075
V-Sh	VM	29.79	27.07	21.83	16.09
	PS	26.74	20.58	19.26	8.52
C-Sh	VM	31.45	27.60	21.77	16.06
	PS	25.44	20.52	19.18	8.49
V-E	VM	29.64	27.70	22.18	16.34
	PS	20.25	20.74	19.51	7.70
C-E	VM	31.26	28.05	22.18	16.34
	PS	20.31	20.75	19.50	7.70

## Data Availability

The original contributions presented in the study are included in the article, further inquiries can be directed to the corresponding author.
